# Global ecological analysis of COVID-19 mortality and comparison between “the East” and “the West”

**DOI:** 10.1038/s41598-022-09286-7

**Published:** 2022-03-28

**Authors:** Ariel Pablos-Méndez, Simone Villa, Maria Cristina Monti, Mario Carlo Raviglione, Hilary Brown Tabish, Timothy Grant Evans, Richard Alan Cash

**Affiliations:** 1 Division of General Medicine, Columbia University Medical Centre, New York, NY USA; 2 Centre for Multidisciplinary Research in Health Science, University of Milan, Via Francesco Sforza, 35, 20122 Milan, Italy; 3 Unit of Biostatistics and Clinical Epidemiology, Department of Public Health, University of Pavia, Pavia, Italy; 4 William Paterson University, Wayne, NJ USA; 5 School of Population and Global Health, McGill University, Montreal, Canada; 6 T.H. Chan School of Public Health, Harvard University, Boston, MA USA

**Keywords:** Health policy, Public health, Epidemiology, Risk factors

## Abstract

Although SARS-CoV-2 was first reported in China and neighbouring countries, the pandemic quickly spread around the globe. This paper explores national drivers of the pandemic and the radically different epidemiology and response in the West and in the East. We studied coronavirus disease (COVID-19) mortality until 31st December 2020, using an ecological study design, considering baseline characteristics and responses that might account for the uneven impact of the pandemic. A multivariable regression model was developed to explore key determinants. Key variables in the West were contrasted with those in the East, and speed of response was examined. Worldwide, 2.24 million COVID-19 deaths were documented in 2020. Western countries reported a median mortality 114 times that of the East (684 vs. 6.0 per million). Significant correlates of mortality in countries with at least 1 million population were median age, obesity prevalence, and democracy index; political stability and experience of SARS in 2002–2003 were protective; health system variables and income inequality were not associated. Outputs of the model were consistent when adjusted for stringency index, timeliness of stay-at-home requirements, and geographical autocorrelation. The West experiences a much higher COVID-19 mortality than the East. Despite structural advantages in the West, delays in national responses early on resulted in a loss of control over the spread of SARS-CoV-2. Although the early success of the East was sustained in the second half of 2020, the region remains extremely vulnerable to COVID-19 until enough people are immunized.

## Introduction

Although the COVID-19 pandemic originated in China and quickly spread throughout Asia, it has had a far greater impact in the western world^1^. Within a few months of the outbreak, the epicentre of the pandemic shifted from Asia to Europe and then to the Americas. In addition to the grim impact on both physical and mental health in Europe and the Americas, lockdowns in *the West* caused wide-spread economic damage, the impact of which is likely to persist for years^2,3^.

During 2020 the COVID-19 mortality rates reported for North America and Europe were 114 times higher than in Western Pacific and South East Asian countries.^11^ The success of *the East* suggests the catastrophe of what ensued in *the West* could have been avoided. The ability of *the East* to prevent and manage SARS-CoV-2 transmission begs the question of how this was possible when advanced economies in *the West*, with established democracies and well-equipped health infrastructure, failed to contain the exponential growth.

This paper explores several potential answers to these questions by reporting on an ecological analysis that compares COVID-19 mortality with baseline country characteristics and national interventions and by focusing on the differences between *the East* and *the West*.

## Methods

We conducted an ecological analysis to describe and investigate the uneven impact of the COVID-19 pandemic in countries with at least 1 million population.

### Data and data sources

The main variable of interest was the cumulative number of reported COVID-19 deaths per million at December 31, 2020, and was retrieved for each observation from *Our World in Data* repository. No human data was analysed.

A large spectrum of variables (supplementary methods and Table S1) was used to explore the possible drivers of reported COVID-19 deaths grouped into three domains:

1. Demographic and health determinants (domain 1);
2. Factors linked with country systemic preparedness and response (domain 2);
3. Structural, economic, and political elements (domain 3).

### Grouping

Countries were arbitrarily grouped into two subsets and labelled as *the West* (i.e., European Union, USA, and Canada) and *the East* (i.e., South-East Asia and the Western Pacific Region of the World Health Organization). The full list of countries is displayed in Table S2. While heterogeneous and evolving, *the East* and *the West* constructs have been used for various socioeconomic, geopolitical and cultural contrasts which framed the inclusion/exclusion criteria before our analysis. The comparison of these regions is relevant for pandemics given both overall levels of public health infrastructure and spending, and the origin (and initial spread) of COVID-19.

To explore the different timing of the adoption of correct behaviours to prevent SARS-CoV-2 infection, we used “face masks” using the relative search volume (RSV) in Google (supplementary materials).

### Statistical plan

Quantitative variables were reported as median and range. COVID-19 deaths per million were logarithmically transformed to normalize the distribution of data. Likewise, gross domestic product (GDP) per capita, population size, and number of air travel passengers were non-normally distributed and consequently logarithmically transformed. All continuous exploratory variables were standardized before their inclusion in the ordinary least squares (OLS) regression models.

The association of the log of COVID-19 deaths per million with likely explanatory variables was further investigated using the Minimum Covariance Determinant (MCD) estimator and regression models with robust standard errors. For multiple OLS regressions, three exploratory sub-models were developed and tested based on specific domains described above. Statistically significant covariates, selected using a stepwise backward selection process, were merged in a unified final exploratory model. The inclusion of interaction terms in the regression models were considered, testing for non-additive effects of different combinations of predictor variables on the dependent variable. Issues of heterogeneity of variance, intragroup correlation and sensitivity to outliers were considered. A model-based clustering and classification estimation was used for corroborating the arbitrary *West* and *East* grouping. Additional information is available in the supplementary materials.

Statistical analysis was performed using STATA version 16.1 (StataCorp, College Station, Texas, USA) or R version 4.0.3 was used for the cluster analysis using the package *mclust*^4^.

## Results

### Demographics and health risk factors

As displayed in Table 1, the global median GDP per capita was 5,152 (range, 369 to 80,504) US dollars in 2019, the estimated proportion of the population aged 65 or older was 6.7% (range, 1.3–28.4%) in 2020, and the median prevalence of obesity was 20.2% (range, 2.1 to 37.9%).

**Table 1:** Demographics, health risk factors, preparedness and response indicators, structural economical-political determinants.

	Median (min; max)	r_MCD_	b (95% CI)^#^
**Demographics**			
Population size (million)	1250 (1–1420)	−0.094^†^	−0.086 (−0.226 to 0.053)^†^
Population density (km^−3^)	83 (2–8358)	0.205	−0.128 (−0.190 to 0.067)*
GPD per capita, USD	5,152 (369–80,504)	0.808^†^	0.512 (0.392 to 0.631)^†^*
Median age (years)	29.6 (15.2–48.4)	0.803	0.509 (0.388–0.631)*
People 65 + years of age (%)	6.7 (1.3–28.4)	0.744	0.480 (0.360–0.600)*
Urban population (%)	60.0 (13.3–100.0)	0.621	0.484 (0.360–0.608)*
Households with 4 + members (%)	52.3 (13.5–93.4)	−0.660	−0.491 (−0.619 to 0.363)*
International migrants (%)	3.0 (0.04–92.9)	0.310	0.167 (0.035–0.298)*
**Health risk factors**			
All−cause mortality (per 1,000 people)	7.2 (1.2–15.4)	−0.413	0.129 (−0.008 to 0.251)*
Obesity prevalence (%)	20.2 (2.1–37.9)	0.674	0.591 (0.476–0.707)*
Prevalence of raised blood glucose (%)	7.7 (4,0–19.6)	0.489	0.098 (−0.037 to 0.232)
Prevalence of raised blood pressure (%)	25.4 (11.0–33.4)	−0.486	−0.267 (−0.413 to 0.121)*
Current tobacco smoking prevalence (%)	24.9 (13.0–39.1)	−0.011	0.442 (0.315 to 0.569)*
BCG immunization coverage (%)			
year 1990	89.5 (13.0–99.0)	0.115	0.035 (−0.161 to 0.232)
year 2019	93.0 (25.0–99.0)	−0.013	0.083 (−0.105 to 0.270)
**Multidimensional preparedness**			
Avg IHR score index (%)	66.0 (17.0–99.0)	0.484	0.379 (0.250–0.507)*
GHS index (%)	41.3 (16.2–83.5)	0.606	0.422 (0.287–0.557)*
UHC service coverage index (%)	68.0 (25.0–89.0)	0.718	0.537 (0.422–0.651)*
Medical doctors (per 10,000 population)	15.68 (0.1–84.2)	0.584	0.493 (0.324–0.662)*
Hospital beds (per 10,000 population)	19.0 (1.0–129.8)	0.482	0.287 (0.096–0.479)*
Previous cases of SARS (‘Yes’ vs. ‘No’)	28 (18.0%)	0.545^§^	−0.037 (−0.523 to 0.450)
**Structural determinants**			
EIU democracy index (0–10)	5.5 (1.1–9.9)	0.421	0.361 (0.232–0.491)*
Variety of democracy (%)	38.0 (9.0–78.0)	0.222	0.277 (0.150–0.404)*
Gini coefficient (%)	36.0 (24.2–63.0)	−0.081	−0.172 (−0.314 to 0.031)*
Literacy rate (%)	91.4 (19.1–100.0)	0.462	0.471 (0.369–0.573)*
Current health expenditure (% GDP)	6.4 (2.1–16.9)	0.515	0.349 (0.192–0.506)*
Government expenditure on essential services (% GDP)	13.8 (5.2–32.5)	−0.227	−0.246 (−0.398 to 0.094)*
Inefficient government bureaucracy (0–30)	10.3 (0.5–23.1)	0.481	0.406 (0.271–0.542)*
Political stability and absence of violence (%)	37.9 (0.0–97.6)	0.175	0.238 (0.095–0.381)*
Government effectiveness index (%)	45.2 (0.0–100.0)	0.403	0.382 (0.247–0.516)*
Air transport, passengers (million)	4.1 (0.0–926.7)	0.223	0.205 (0.040–0.372)*
Island countries (‘Yes’ vs. ‘No’)	18 (11.5%)	0.079^§^	−0.367 (−0.779 to 0.044)

Robust correlation coefficient (r_MCD_) based on the Minimum Covariance Determinant estimator was computed to estimate the strength of the association between the covariate and the log of COVID-19 deaths per million. * Significant p-values for b coefficients with robust standard errors of the simple OLS regression models against COVID-19 deaths per million (logged). ^#^ Covariates were standardized before computing OLS regression coefficients. ^†^ Log-transformed to normalize the distribution frequency. ^§^ Not significant student t-value calculated to compare log of COVID-19 mortality between countries with and without previous SARS cases.

*BCG* Bacillus Calmette-Guerin; *COVID-19*   coronavirus disease 2019; EIU   Economist intelligence unit; *GDP*   gross domestic product; *GHS*   Global Health Security; *IHR*   International Health Regulations; *MCD*   Minimum Covariance Determinant; *SARS*   Severe acute respiratory syndrome; UHC = universal health coverage.

There was a positive and strong association between the log of GDP per capita and the log of COVID-19 mortality (MCD = 0.808, *b* = 0.512, *p* < 0.001). Likewise, age was associated with increased mortality both as median age (MCD = 0.803, *b* = 0.509, *p* < 0.001) and as proportion of population 65 + years of age (MCD = 0.744, *b* = 0.480, *p* < 0.001). While a higher proportion of the population living in urban settings was positively associated to COVID-19 mortality (MCD = 0.621, *b* = 0.484, *p* < 0.001), living in crowded households had an inverse association (MCD = -0.660, *b* = −0.491, *p* < 0.001).

Several health risks beyond age have been associated with poor COVID-19 outcomes. Prevalence of obesity, in particular, had a strong positive association with COVID-19 mortality (MCD = 0.674, *b* = 0.591, *p* < 0.001) followed by while the prevalence of raised blood pressure had a strong negative association with COVID-19 mortality (MCD = −0.486, *b* = −0.267, *p* < 0.001).

### Health system preparedness and response indicators

Several preparedness indicators were significantly associated with COVID-19 mortality (Table 2). In particular, the service coverage index of universal health coverage (UHC) was positively associated with COVID-19 mortality (MCD = 0.718, *b* = 0.537, *p* = 0.002), as was the density of medical doctors (MCD = 0.584, *b* = 0.493, *p* < 0.001). Notably, scores used specifically to evaluate countries’ preparedness, like the average score from the country self-reporting preparedness within the 2005 International Health Regulations (IHR) (MCD = 0.484, *b* = 0.379, *p* < 0.001) and the global health security (GHS) index (MCD = 0.606, *b* = 0.422, *p* < 0.001), were also associated with increased COVID-19 mortality.

**Table 2:** Final multivariate model for global COVID-19 mortality adjusted for response measures.

	Final model	Correction for spatial autocorrelation
	b (95% CI) *p*-value	b (95% CI) *p*-value
Constant	1.963 (1.871–2.055) *p* < 0.001	1.963 (1.869–2.057) *p* < 0.001
Median age (years)	0.370 (0.238–0.501) *p* < 0.001	0.370 (0.145–0.594) *p* = 0.004
Obesity prevalence (%)	0.358 (0.260–0.455) *p* < 0.001	0.358 (0.189–0.526) *p* < 0.001
Previous cases of SARS (‘Yes’)	−0.311 (−0.552–−0.069) *p* = 0.012	−0.311 (−0.712 to 0.090) *p* = 0.118
EIU democracy index (0–10)	0.353 (0.232 to 0.475) *p* < 0.001	0.353 (0.147 to 0.560) *p* = 0.003
Political Stability and Absence of Violence (%)	−0.297 (−0.428 to −0.166) *p* < 0.001	−0.297 (−0.481 to −0.113) *p* = 0.004
Average stringency index (%)	0.160 (0.052 to 0.268) p = 0.004	0.160 (0.035 to 0.285) *p* = 0.016
Timeliness of stay−at−home requirements (days)	−0.035 (−0.129 to 0.059) *p* = 0.458	−0.035 (−0.155 to 0.085) *p* = 0.537
**Model statistics**		
Observations	133	
R^2^	0.686	
Adjusted R^2^	0.669	
Residual SE	0.476	
F statistic (df)	39.05 (7, 125)	123.60 (7, 13)
p−value	p < 0.001	
AIC	187.79	
Sample−size adj. BIC	210.91	

In the final model, three observation (ie, Mongolia, Thailand, and Papua New Guinea) was removed because outlier, as described in the supplementary results. Regions are controlled as sampling clusters and the clustering effect was accounted for using cluster-robust standard errors (the clustered sandwich estimator) .

*EIU* Economist intelligence unit; *SARS* Severe acute respiratory syndrome.

### Structural socio-political determinants

The proportion of literate population was strongly associated with COVID-19 mortality (MCD = 0.462, *b* = 0.471, *p* < 0.001), as were the level of inefficient government bureaucracy (MCD = 0.481, *b* = 0.406, *p* < 0.001) and government effectiveness index (MCD = 0.403, *b* = 0.382, *p* < 0.001) (Table 2). Negative associations with COVID-19 mortality were notable for the proportion of GDP used on essential services (MCD = −0.227, *b* = −0.246, *p* = 0.002) and the Gini coefficient (r = −0.081, *b* = −0.172, *p* = 0.018).

### Exploratory multivariable models

**Figure 1:**
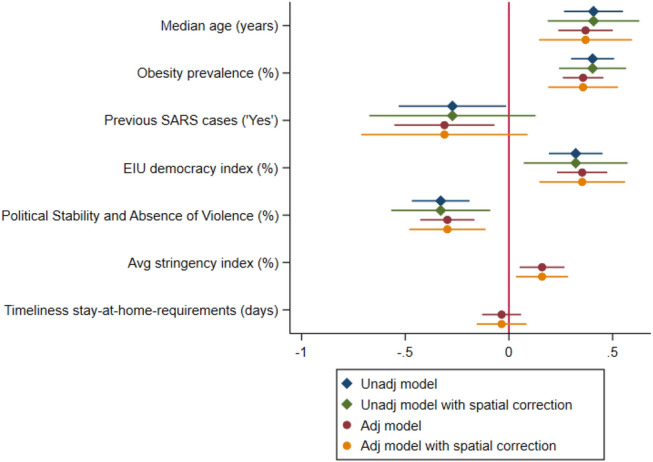
Forest plot of the final multivariate OLS regression model of COVID-19 mortality in 2020 adjusted for response measures, with spatial autocorrelation correction. In the figure, three countries (ie, Mongolia, Thailand, Papua New Guinea) were removed because outliers. Additional information can be found in the supplementary results.

We developed three domain-specific OLS regression sub-models (Table S4). Only five variables were found to be associated with COVID-19 mortality in the final multivariate model (Table S5 and Fig. 1) and the significance of the outputs remained unchanged once adjusted for response measures (Table S6). The final adjusted OLS model, further refined by removing outliers as described in the supplementary results and in Figure S6, is reported in Table 2. In this, increased COVID-19 deaths per million are associated with higher median age (*b* = 0.370, *p* < 0.001), higher prevalence of obesity in adults (*b* = 0.358, *p* < 0.001), and higher Economist’s Intelligence Unit (EIU) democracy index (*b* = 0.353, *p* < 0.001). On the other hand, previous local SARS outbreaks (*b* = −0.311, *p* = 0.001), and the index for political stability (*b* = −0.297, *p* < 0.001) emerged as protective factors against COVID-19 mortality. The significance of the model was further tested for the geographical autocorrelation with only previous SARS cases losing its significance (Fig. 1).

#### Comparing the East versus the West

**Figure 2:**
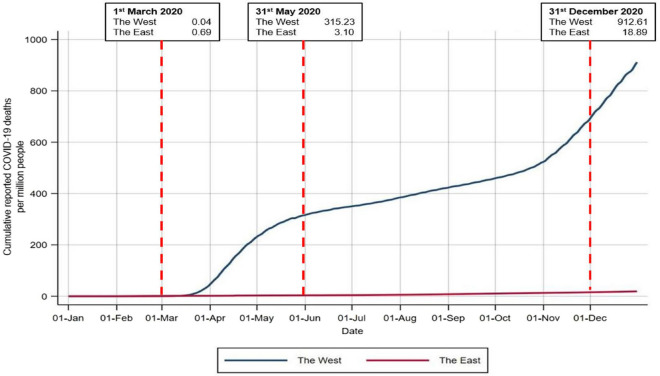
Trends in COVID-19 mortality (per 1 million people) in the West and in the East. Displayed mortality rates are unstandardized as data disaggregated by age groups are not available in any public repository. All countries displayed in this figure are those listed in Table S2 of the supplementary methods, including those < 1 million population.

On average, the log of COVID-19 mortality in *the West* compared to *the East* increase of 2 units using a univariate OLS model with robust standard errors (*b* = 2.012, robust 95%CI: 1.535–2.488, *p* < 0.001). The median COVID-19 mortality reported in *the West* was 68.4 per million, much higher than the median in *the East* 6.0. Among Western countries, Belgium had by far the highest mortality (1710.1 per million population) and Norway the lowest (81.5 per million). In *the East* the highest rates were registered in Indonesia (85.3 per million), the Philippines (82.4 per million), and Myanmar (49.1 per million population). Figure 2 displays the trends of COVID-19 mortality in the two regions throughout the 2020.

*The West* had a median GDP per capita 8-times higher than *the East*, and their populations were older (median age 43.2 vs. 31.4 years, respectively)(Table 3). Western countries were more urbanized, while *the East* had a higher proportion of households with 4 or more members (median 58.6 vs. 22.2%). The prevalence of obesity was much higher in *the West* (median 23.1%) than in *the East* (6.2%), and only slightly more so for smoking.

**Table 3:** Demographics, health risk factors, preparedness and response indicators, structural economical-political determinants within the two regions of interest.

	The West	The East	p-value
	Median (min; max)	Median (min; max)	
**Demographics**			
Population size (million)	10 (1–328)*	28 (13–1420)	0.084
Population density (km^-3^)	100.0 (4.0–508.2)*	151.0 (2.0–8,358)	0.089
GPD per capita, USD	33,159 (9772–80,504)	4,202 (1,252–65,234)	< 0.001*
Median age (years)	43.2 (38.3–47.3)	31.4 (20.8–48.4)	< 0.001*
People 65 + years of age (%)	20.2 (14.6–23.3)	7.53 (3.6–28.4)	< 0.001*
Urban population (%)	75.4 (53.7–98.0)	53.3 (13.3–100.0)	0.019*
Households with 4 + members (%)	22.2 (13.5–32.1)	58.6 (20.0–83.8)	< 0.001*
International migrants (%)	12.5 (1.7–21.8)	0.6 (0.1–38.0)	< 0.001*
**Health risk factors**			
All-cause mortality (per 1,000)	10.5 (6.4–15.4)	6.3 (5.0–11.0)	< 0.001*
Obesity prevalence (%)	23.1 (19.7–36.2)	6.2 (2.1–29.0)	< 0.001*
Prevalence of raised blood glucose (%)	6.6 (4.3–7.9)	7.7 (5.3–14.8)	0.002*
Prevalence of raised blood pressure (%)	21.0 (12.9–32.4)	23.2 (11.0–29.0)	0.751
Current tobacco smoking prevalence (%)	27.1 (13.0–39.1)	22.0 (14.1–38.2)	0.101
BCG immunization coverage (%)			
year 1990	89 (13–99)	91 (26–99)	0.919
year 2019	96 (25–99)	88 (69–99)	0.563
**Multidimensional preparedness**			
Avg IHR score index (%)	82.0 (57.0–99.0)	73.0 (21.0–97.0)	0.276
GHS index (%)	60.3 (45.6–83.5)	49.3 (26.0–75.5)	0.029*
UHC service coverage index (%)	78.0 (66.0–89.0)	67.5 (40.0–87.0)	0.032*
Medical doctors (per 10,000 population)	36.1 (23.1–63.5)	8.2 (0.7–36.8)	< 0.001*
Hospital beds (per 10,000 population)	45.7 (21.4–80.0)	24.9 (9.0–129.8)	0.075
Previous cases of SARS (‘Yes’ vs. ‘No’)	10 (37.0%)	12 (66.7%)	0.099
**Structural determinants**			
EIU democracy index (0–10)	8.0 (6.5–9.9)	6.5 (2.1–9.1)	< 0.001*
Variety of democracy (%)	71.0 (29.0–78.0)	44.0 (14.0–77.0)	0.002
Gini coefficient (%)	31.6 (24.9–41.1)	35.7 (28.7–44.4)	0.020
Literacy rate (%)	99.1 (95.3–99.9)	94.5 (63.4–98.4)	< 0.001*
Current health expenditure (% GDP)	9.0 (5.6–16.9)	4.4 (2.3–11.0)	< 0.001*
Government expenditure on essential services (% GDP)	12.1 (7.8–16.0)	13.2 (7.9–28.8)	0.255
Inefficient government bureaucracy (0–30)	12.9 (6.0–21.8)	10.7 (3.1–19.7)	0.066
Political stability and absence of violence (%)	71.4 (57.1–92.4)	52.4 (11.4–97.6)	< 0.001*
Government effectiveness index (%)	84.1 (40.4–99.0)	63.0 (11.5–8.8)	0.003*
Air transport, passengers (million)	18.7 (0.0–926.7)	50.5 (0.7–659.6)	0.582
Island countries (‘Yes’)	2 (7.4%)	6 (33.3%)	0.069
**Response measures**			
Avg stringency index (%)	48.5 (32.7–59.8)	51.1 (24.0–68.3)	0.828
Timeliness of stay-at-home requirements (days)	27 (6–254)	45 (2–190)	0.173

P-values reported were computed with Mann–Whitney U test or Pearson’s chi-squared test between the West and the East.

*BCG* Bacillus Calmette-Guerin; *COVID-19* coronavirus disease 2019; *EIU* Economist intelligence unit; *GDP* gross domestic product; *GHS* Global Health Security; *IHR* International Health Regulations; *SARS* Severe acute respiratory syndrome; *UHC* universal health coverage.

*The West* had significantly better indicators of pandemic preparedness (ie, IHR and GHS indices), health system performance (ie, density of medical doctors, hospital beds), and health spending. Two thirds of the countries in *the East* experienced SARS in 2002–2003 compared to 37% in *the West*. Western countries had better indicators of governance, literacy and income inequality, and were more likely to enjoy political stability free of violence compared to *the East*. Lastly, of the countries included in this analysis more countries in *the East* were islands than in *the West* (33.3% vs. 7.4%).

When analysing how the subset of countries clustered based on the Table 2 features, we corroborated the empirical *East–West* classification. However, countries of the Eastern Europe were observed to form a distinct cluster compared to countries of the Western Europe and North America. (details and plots in the supplementary results).

### Timeliness and the maintenance of response measures to COVID-19

As shown in Fig. 2, the cumulative mortality from COVID-19 in *the West* skyrocketed nearly 8000-fold from March 1st to May 31st, but only 4.5 times in *the East*; from May 31st to December 31^st^ the relative increases were 2.9 and 6.0, respectively.

*The East* and *the West* had a similar stringency index for their response to COVID-19, but *the West* was delayed and more reactive and it failed to halt an early and catastrophic exponential rise in cases and deaths (Table 4). The effective reproduction rate for COVID-19 was higher in the first quarter of 2020, and more so in *the West* vs. *the East*; the opposite was true for the stringency of the response. The positive rate of tests performed was much higher in *the West*.

**Table 4:** COVID-19 Epidemiology and response during 2020.

	World	The West	The East	*p*-value
	median (range)	median (range)	median (range)	
**COVID-19 reproduction rate (Re)**^**§**^				
Q1	1.7 (0.2–5.8)	2.0 (1.0–3.7)	1.5 (0.2–5.8)	< 0.001
Q2	1.1 (0.0–4.0)	0.9 (0.1–3.3)	0.8 (0.0–2.3)	< 0.001
Q3	1.0 (0.0–3.0)	1.2 (0.5–2.4)	0.9 (0.2–3.0)	< 0.001
Q4	1.0 (0.0–3.3)	1.1 (0.6–2.0)	1.0 (0.3–1.8)	< 0.001
**Stringency Index (%)**				
Q1	33.3 (0.0–100.0)	20.4 (0.0–96.3)	31.5 (0.0–100.0)	0.002
Q2	76.8 (9.3–100.0)	70.8 (25.9–96.3)	68.1 (22.2–100.0)	< 0.001
Q3	60.2 (11.1–100.0)	48.2 (23.1–76.4)	52.8 (20.4–83.3)	< 0.001
Q4	55.6 (6.5–89.8)	62.0 (23.1–84.3)	50.0 (19.4–85.2)	< 0.001
**Total tests (per 1000 population)**				
Q1	0.1 (0.0–22.2)	0.7 (0.0–11.2)	0.1 (0.0–12.9)	< 0.001
Q2	7.0 (0.0–363.3)	28.4 (0.7–183.8)	3.1 (0.0–129.5)	< 0.001
Q3	36.1 (0.7–981.2)	116.4 (19.6–671.4)	13.2 (1.4–493.2)	< 0.001
Q4	90.8 (2.5–2112.2)	307.8 (75.3–1819.7)	34.8 (3.9–927.5)	< 0.001
**Test per case**				
Q1	22.7 (1.8–5951.0)	15.2 (3.1–202.8)	40.2 (2.8–1876.5)	< 0.001
Q2	30.8 (1.8–44,258.7)	56.9 (2.3–5097.9)	127.8 (2.4–44,258.7)	< 0.001
Q3	20.8 (1.6–37,002.3)	67.0 (7.9–1443.8)	231.3 (4.2–37,002.3)	< 0.001
Q4	13.4 (2.0–9601.7)	11.3 (2.0–122.8)	72.4 (4.6–9601.7)	< 0.001

^§^COVID-19 Reproduction rate (Re) figures are the median values of the quarter within the region of interest, either the West or the East.

A Mann–Whitney U test was used to test for differences between the West and the East. We had also performed a non-parametric test for trend across quarters (data not shown): *p*-values were < 0.001 for the World, the West and the East for all response variables expect for stringency index in the East (*p*-value = 0.007).

**Figure 3:**
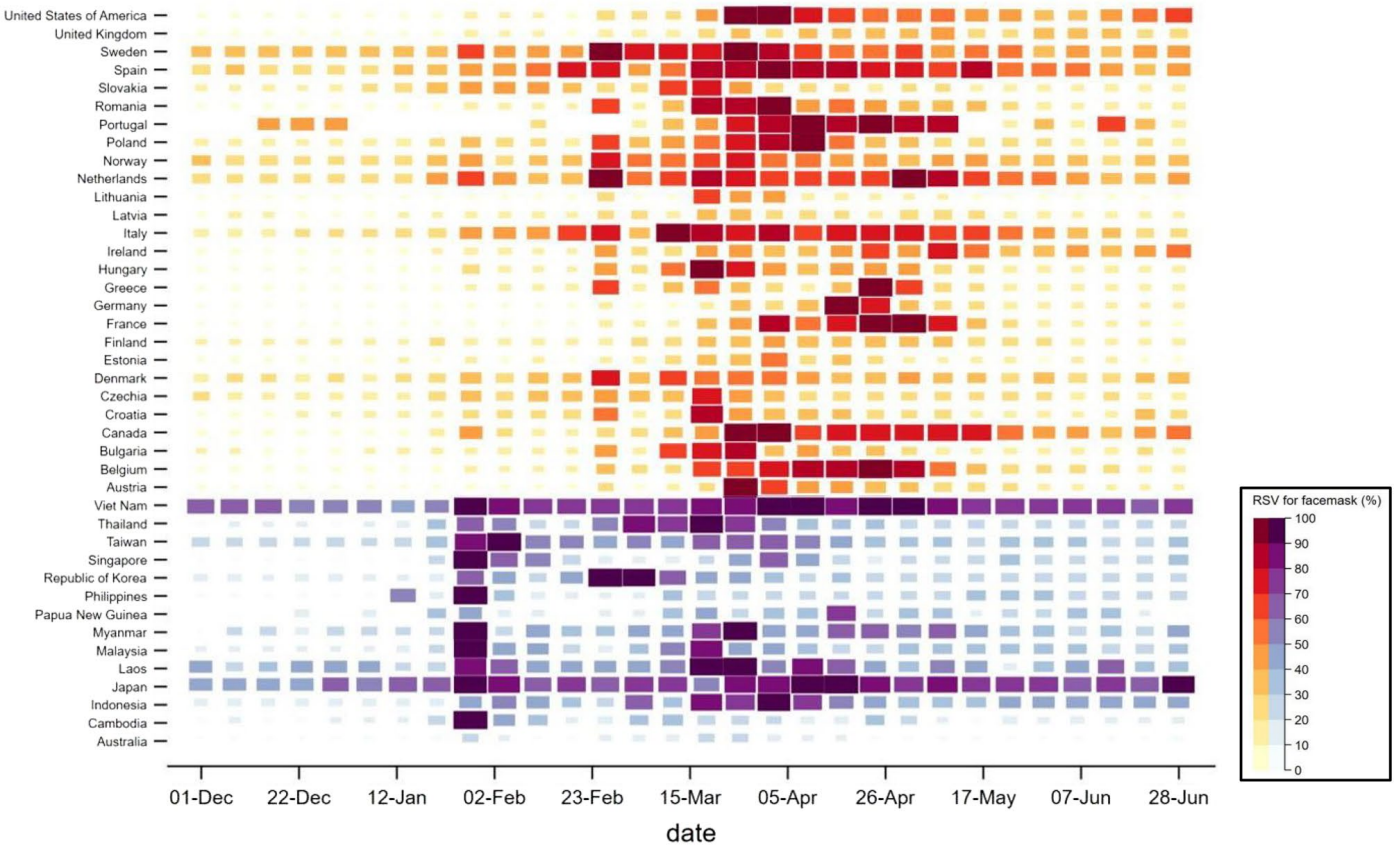
Weekly trend in online searches of “face masks” in Google, expressed in RSV (%), in the West (in red) and in the East (in purple). As extensively explained in the supplementary materials, data from 1st December 2019 to 30th June 2020, accounting for 1-month period of baseline to account for possible confounding for some countries in the East with higher baseline values (ie, Japan and Viet Nam). Additional information on Google Trend RSV can be found in the supplementary methods.

In *the East* internet users increased searches for masks at the end of January, while *the West* such web-searches were delayed until March–April (Fig. 3). The temporal delay persists after adjusting for the date of each country’s first confirmed case.

## Discussion

In 2020, there were 2.24 million confirmed COVID-19 deaths reported worldwide and this figure is estimated to surpass 10 million in reality^5^. Significant elements positively associated with increased COVID-19 mortality were median age, obesity prevalence, and EIU democracy index; political stability and previous SARS cases were associated with lower mortality. Despite having robust economies and health systems, Western countries experienced a COVID-19 mortality 114-times higher than countries in *the East*. Aging^5^ and obesity^6^, well established risk factors for COVID-19 severity, were more prevalent in *the West* and may have contributed to the higher mortality there. Having experienced SARS, on the other hand, seemed protective and may have helped *the East* mount a swifter response.

Disappointingly, the GHS index, the average score of IHR self-assessment, UHC, and the density of both hospital beds and medical doctors did not seem to protect against COVID-19 mortality. Structural factors played a less significant role at the global level, including the Gini coefficient which has been established as important at the subnational level^7^. The index used to measure political stability was associated with lower COVID-19 mortality, while, the EIU democracy index was associated with worse COVID-19 mortality; this suggests that authoritarian regimes with command-and-control leadership and more pliable societies may have been more effective in containing the epidemic^8^.

Delays in responding to the pandemic may have played a large role in the health and social crisis. The reproduction number of COVID-19 was much higher in *the West* in the first quarter of 2020 and cumulative mortality skyrocketed nearly 8,000-fold from March 1^st^ to May 31^st^, but rose less than tenfold in *the East* and from May 31^st^ to December 31^st^. During the first quarter of 2020, Asian countries tested 2.7 times more patients per reported COVID-19 case than in Western countries^9^. Testing is paramount not only for surveillance but for early contact-tracing and prompt isolation^10^—time-honoured public health measures that South Korea and other neighbouring countries implemented early and with rigor. Similarly, the use of face masks was more prevalent in Asia^11^ and interest in them surged several weeks before they did in *the West*. In addition to masks becoming politicized in *the West*^12^, their slow uptake in this region may have been due to the WHO’s slow recognition of the effectiveness of masks and therefore lack of leadership in this area^13^.

Interestingly, countries that reported local SARS outbreaks in 2002–2003 had a lower COVID-19 mortality in 2020. While the viruses causing SARS and COVID-19 are closely related, protective cross-immunity in 2020 is improbable because of the very limited number of SARS cases and relatively short duration of neutralizing antibodies^14^. More plausible, the “social memory” derived from experiencing a highly lethal outbreak and associated economic shock, especially in Asia, may have contributed to a faster and consistent response at individual and community levels. Conversely *the West,* with more robust economies and stronger health systems, may have been overconfident causing delays in response and losing control of the epidemic early on which ultimately required more drastic countermeasures^15^. The costs of lock-downs will persist over several years and will impact both the political economy as well as the physical and mental health of the population.

In the summer of 2020, there was concern that the lack of population-level immunity and exhaustion with strict behavioural measures would lead to a spike of COVID-19 deaths in *the East* by the end of the year like the one experienced by *the West* in the spring. Instead, *the West* missed a second chance at controlling the pandemic^16^ and experienced an even greater wave (in absolute numbers) by the end of 2020. This included Sweden, whose more liberal (and sustainable) policies permitted the un-interrupted transmission of the virus among young and healthy people^17^. A sustained response to COVID-19 was clearly more important than slowly growing levels of natural immunity^18^.

With much lower levels of natural immunity in 2021^19^, Asian populations remain very vulnerable to COVID-19 as documented by the rapidly increasing burden in some countries in the first half of 2021. This is a clear signal that these countries should not lower their guard until enough people are vaccinated, starting with the elderly, health care workers and other vulnerable populations. The wave of cases in India in early 2021 is a case in point, as is the recent increase in mortality rates in Taiwan, Japan and Thailand.^11^ The prognosis in Africa, also reporting low COVID-19 mortality in 2020, is less clear given its younger population^20^.

This study has some limitations as data from different sources of data are far from perfect nor complete and do not disaggregate by key elements such as age or socioeconomic status. Further, numerous COVID-19 cases, as well as deaths, likely went undetected or unreported which may have been more prevalent in the East.^20^ Noteworthy, different types of definitions were used by countries to define a COVID-19 death, either using the current WHO definition (i.e., clinical diagnosis-based for confirmed and probable cases)^21^ or a test-based diagnosis^22^. To attenuate this challenge our analysis focused on mortality rather than incidence. We did not analyze the importance of culture, climate change or biological indicators (genetics, mutations), although we note that in the United States persons of Asian descent had the same COVID-19 mortality as non-Hispanic Whites^23^. Several paradoxical findings in univariate analyses, like higher mortality in countries with better health preparedness indicators, may have been confounded by higher case detection or, more likely, by delays activating their public health capability before contagion spun out of control. Furthermore, as described with the availability and reliability of data on COVID-19 epidemiology, other predictors used in the models might be affected by the same under-estimation (e.g. obesity prevalence and median age). These, however, are estimated from international organizations, while COVID-19 cases and deaths are those directly reported by countries and not based on estimates accounting for under-reporting.

## Conclusion

*The East* was successful in stopping the exponential first phase of COVID-19 in 2020, while *the West* has suffered mortality rates 114 times higher. Older age and obesity emerged as significant ecological risk factors for COVID-19 and might have contributed to the vast differences between these two regions. Rapid response from governments, including early lock-downs in China, higher testing coverage per case in South Korea, and early mask wearing in most of Asia may have contributed to the East’s ability to contain the epidemic. Similarly, countries that experienced SARS in 2002–2003 had lower deaths from COVID-19 in 2020^24^. Whereas delays in response and ineffective leadership and communication hindered western countries’ ability to control the virus, despite their stronger economies and more robust health systems ^25,26^, While the early success of *the East* was sustained throughout 2020, low natural immunity in the region requires continued public health vigilance until threshold levels of vaccination-acquired immunity are reached or the virus otherwise disappears from the face of the Earth.

## Supplementary Information

Supplementary Information.
