# Supplementary material for: Global ecological analysis of COVID-19 mortality and comparison between “the East” and “the West”
Source: Sci Rep. 2022 Mar 28;12:5272. doi: 10.1038/s41598-022-09286-7 (PMC8959561; doi:10.1038/s41598-022-09286-7)
Supplement: Supplementary file 1 — Supplementary Information. [file 41598_2022_9286_MOESM1_ESM.docx]

**Supplementary materials**

**Global Ecological Analysis of COVID-19 Mortality and Comparison Between “the East” and “the West”**

**Table of content**

[Supplemental Methods 2](#_Toc74136408)

[Supplemental Results 6](#_Toc74136409)

[Supplemental Tables 8](#_Toc74136410)

[Supplemental Figures 14](#_Toc74136411)

[Post-Estimation Diagnostics And Robustness Checks 19](#_Toc74136412)

# **Supplemental Methods**

**Measurement of face covering behaviours**

Having no publicly-available, robust, and standardized repository to collect data either about face masks stocks or behaviour for mask wearing in the first months of the pandemic and accounting for a baseline scenario (pre-COVID-19 pandemic), we have decided to rely on the relative search volume (RSV) for “face mask” from Google Trend®.

The RSV represents the unfiltered sample of search requests made to Google® and stored anonymized and aggregated, based on location (e.g. by country). Repeated searches from the same user over a short period of time are eliminated from RSV trends, to avoid overestimation. This index ranges from 0 to 100 based on the number of searches in a specified period of time. Therefore, we extended the period from 1^st^ December 2019 to 30^th^ June 2020, to include a baseline period pre-COVID-19 (i.e., from 1^st^ to 31^st^ December 2019). Because of the limitations imposed to the use of Google, RSV was not gathered for China. Data was also instable and scarce for Timor-Leste; therefore, it was not possible to extract RSV data for the country.

We used RSV for “face masks” as a proxy of individuals (i.e., internet users) willingness to be informed on (i) where to buy, (ii) buy on-line, and/or (iii) how to use, face masks; ultimately, RSV can be therefore seen as an element expressing the adoption of other physical distancing measures (e.g. avoid crowded places if face masks are not available). Because the fast implementation, also at individual-level, is essential in preventing further spread of SARS-CoV-2 we focused our attention for this parameter in the “first wave” (i.e., until 30^th^ June 2020), after which the pandemic irreversibly spread and entered into its cyclic phase. Moreover, after the early phases of the pandemic, RSV value reduction can correspond, for example, to a more capillary accessibility of surgical masks or other type of masks in the community and increase knowledge and awareness of the population on their use.

**Adjusting for response measures**

Throughout the year 2020, governments have implemented a vast number of non-pharmacological interventions (NPIs) from mandatory face covering measures to stay-at-home requirements and lock-downs, thus resulting in major direct and indirect health and socio-economic consequences. The final model we developed with baseline characteristics was, therefore, lacking of these measures of response against COVID-19. However, due to the complexity in summarize such a vast range of measures implemented at local, national, and regional level, we preferred to rely to a common multidimensional measure developed by the Oxford Covid-19 Government Response Tracker (OxCGRT). The stringency index is a composite indicator resulting from the summary of containment and closure policies implemented (i.e., school closing, workplace closing, public events cancelled, restrictions on gatherings, closed public transport, stay-at-home requirements, restrictions on internal movements, and international travel controls) and public information campaigns.

The timeliness of implementation of stay-at-home requirements was computed using data reported in the OxCGRT repository as categorical variables and saving the date of the first recording the requirement of not leaving house with minimal exceptions (labelled as “3” in the OxCGRT repository). The variable regarding the timeliness of stay-at-home requirements was then computed from the date of the first COVID-19 case detection per each country and the date of the implementation of such requirements.

The final OLS model, resulting from the stepwise selection process of merged sub-models (one for each domain), was adjusted for two response measures: (i) timeliness of implementation of stay-at-home requirements (days) and (ii) average value of stringency index (%). Sources of data for both variables are available in **Table S1**. Both variables were standardized before entering in the final OLS regression model.

Data concerning COVID-19 tests, either the number of tests per thousand nor test per confirmed COVID-19 case or the proportion of positive tests, were not incorporated in the OLS modelling because of the high number of countries with missing information.

**Adjusting for the day of 1^st^ COVID-19 detection**

We developed additional models accounting for the date of the detection and reporting of the 1^st^ COVID-19 case. Data were retrieved from OWID (**Table S1**) and days from the 1^st^ case reported and the censoring date (i.e., 31^st^ December 2020) were computed and logarithmically transformed. Prior being included in the regression models, the variable was standardized.

**Adjusting for the COVID-19 tests’ positive rate**

Data concerning the positive rate to COVID-19 tests reported by countries at 31^st^ March 2020 were retrieved from *Our World in Data* (see **Table S1**). This information is, however, only available for 61 countries whose population is greater than 1 million people.

**Checking the issues of heterogeneity of variance, intragroup correlation and the sensitivity to outliers of the linear OLS models**

In the linear models, standard errors allowing for intragroup correlation were calculated (using the clustered sandwich estimator) to take account for the nested effect of geographical regions. A graphical comparison by forest plot was made to show the standard errors resulting from linear OLS regression models with and without the clustered sandwich estimator of the variance (**Figure 2**). Moreover, the issue of heterogeneity of variance was explored comparing robust standard errors (using the Huber sandwich estimator) with the regular ones. Standard, clustered-robust, and robust standard errors were compared (see **Tables S5** and **S6** in the **supplemental results**).

To check the sensitivity of OLS models to outliers and, therefore, to better reflect the relationship between the COVID-19 mortality and the predictor variables seen in the rest of the records, a robust regression model was also applied and the final weights assigned to the countries were discussed. A graphical comparison by forest plot was made to show the coefficients and standard errors resulting from analogous linear OLS regression models and robust regression models. Some countries were considered influential outliers (considering also Grubb’s method) and, therefore, they were excluded from the final dataset (see the paragraph “Identification and exclusion of influential outliers” in the supplemental results).

**Statistics for *West* vs. *East* comparison**

To compare the two regions, defined as reported in **Table S2**, a violin plot was used to show countries mortality and the kernel probability density of the mortality data according to the region of interest (**Figure S1**, produced with the ggplot2 package of the software R version 4.0.3) while the mortality trend over 2020 in the two regions is shown in Figure 2.

A non-parametric Mann-Whitney U test was used for comparing continuous variables, while a Chi-squared test was computed for categorical variables in **Table 3**. Data for response measures implemented, as displayed in Table 4, were computed using the median value of each observation during each quarter of year 2020 and, then, used to calculate a region-specific median value. A Mann-Whitney *U* test was utilized to compute p-values between the two regions of interest, while, a non-parametric test for trends across quarters was used to assess significance within the two regions (Conover, W. J. 1999. Practical Nonparametric Statistics, 3rd ed. New York: Wiley; Cuzick, J. 1985. A Wilcoxon-type test for trend. Statistics in Medicine 4: 87-90).

**Cluster analysis**

To corroborate the empirical grouping of the countries selected to define *the West* and *the East* regions (as listed in in **Table S2**), we employed a model-based clustering and classification estimation based on finite Gaussian mixture modelling by using the *mclust* and *factoextra* R packages (version 4.0.3). The mortality predictors selected in the final multivariable OLS model (**Table 2**) were used for the cluster analysis (i.e., countries in **Table S2**). The optimal number of clusters was selected based on the Bayesian Information Criterion (BIC) (**Figure S9a**).

***Table S1* Set of variables used**

| **Variable** | **Year** | **Source** | **URL** |
| --- | --- | --- | --- |
| Population size | 2020 | UNDESA | <https://bit.ly/2Pi2ZYt> |
| Population density (pop/km^3^) | 2020 | UNDESA | <https://bit.ly/2Pi2ZYt> |
| Median age (years) | 2020 | UNDESA | <https://bit.ly/2Pi2ZYt> |
| Population 65+ years of age (%) | 2020 | UNDESA | <https://bit.ly/2Pi2ZYt> |
| Urban population (%) | 2019 | World Bank | <https://bit.ly/2PuS39I> |
| Households 4+ members (%) | * | UNDESA | <https://bit.ly/3rMf1q2> |
| International migrants (%) | 2015 | World Bank | <https://bit.ly/2QYGhoF> |
| All-cause mortality (per 1,000 pop) | 2018 | World Bank | <https://bit.ly/3cIGjJS> |
| GDP per capita (current USD) | 2019 | World Bank | <https://bit.ly/3dntIuI> |
| Obesity prevalence (%) | 2016 | WHO-GHO | <https://bit.ly/39RgvcH> |
| Raised blood glucose prevalence (%) | 2014 | WHO-GHO | <https://bit.ly/39CrVR8> |
| Raised blood pressure prevalence (%) | 2015 | WHO-GHO | <https://bit.ly/3cJmlOM> |
| Tobacco smoking prevalence (%) | 2018 | WHO-GHO | <https://bit.ly/2QTjLNL> |
| BCG immunization coverage (%) | 1990, 2019 | WHO-GHO | <https://bit.ly/3fAsSgS> |
| UHC service coverage (%) | 2017 | SDG | <https://bit.ly/3fvusAB> |
| IHR average score | 2019 | WHO-GHO | <https://bit.ly/39BbCUJ> |
| GHS index (%) | 2019 | GHS | <https://bit.ly/3sDDzCV> |
| Timeliness of stay-at-home requirements | 2020 | OxCGRT | <https://bit.ly/31HonJb> |
| Average stringency index (%) | 2020 | OxCGRT | <https://bit.ly/31HonJb> |
| Test per COVID-19 case | 2020 | OWID | <https://bit.ly/3maKlgU> |
| Medical doctors (per 10,000 pop) | * | WHO-GHO | <https://bit.ly/3sIFTbD> |
| Hospital beds (per 10,000 pop) | * | WHO-GHO | <https://bit.ly/3mefiBa> |
| EIU democracy index (0-10) | 2019 | EIU | <https://bit.ly/31RVyKh> |
| Varieties of Democracy (%) | 2019 | V-dem | <https://bit.ly/39xMLkG> |
| Inefficient government bureaucracy (0-30) | 2017 | World Bank | <https://bit.ly/3mko4O0> |
| Government effectiveness index (%) | 2017 | World Bank | <https://bit.ly/2OeORP9> |
| Gini index (%) | 2019 | World Bank | <https://bit.ly/2PjJ2AA> |
| Literacy rate (%) | 2015 | OWID | <https://bit.ly/3dsXazq> |
| Government spending on essential services | * | SDG | <https://bit.ly/3fvusAB> |
| Air travel passengers (million) | 2019 | World Bank | <https://bit.ly/3dplUZb> |
| Island countries | - | Wikipedia | <https://bit.ly/3dvM4d3> |
| Previous isolation of SARS cases | 2003 | WHO | <https://bit.ly/3rFX4JS> |
| Date of 1^st^ COVID-19 case reported | 2020 | OWID | <https://bit.ly/3maKlgU> |
| RSV for “face masks”^§^ | 2020 | Google | <https://bit.ly/3ufoFmE> |

* Latest available data for each observation
^§^ Data was manually retrieved

***Table S2* List of countries selected to define the West and the East**

| **The** **West** (n = 31) | Austria  Belgium  Bulgaria  Canada  Croatia  Cyprus^*^  Czech Republic  Denmark  Estonia  Finland  France  Germany  Greece  Hungary  Iceland^*^  Ireland  Italy  Latvia  Lithuania  Luxembourg^*^  Malta^*^  Netherland  Norway  Poland  Portugal  Romania  Slovakia  Spain  Sweden  United Kingdom  United States of America | **The East**  (n = 25) | Australia  Brunei Darussalam^*^  Cambodia^§^  China  Fiji^*^  Hong Kong  Indonesia  Japan  Laos^§^  Malaysia  Marshall Islands^*§^  Mongolia  Myanmar  New Zealand  Papua New Guinea  Philippines  Republic of Korea  Samoa^*^  Singapore  Solomon Islands^*§^  Taiwan  Thailand  Timor-Leste^§^  Vanuatu^*§^  Viet Nam |
| --- | --- | --- | --- |

^*^ Population < 1 million people and therefore excluded from the analysis

^§^ No deaths reported, thus dropped when COVID-19 deaths per million were log transformed.

# **Supplemental Results**

A full list of the countries (n=156) included in the multivariable OLS regression modelling is reported in **Table S3**, for which COVID-19 incidence (cases per million population), mortality (deaths per million population), and case-fatality ratio (% deaths among cases) at December 31, 2020, are displayed.

**Robustness Check Accounting for the Nested Effect of Geographic Regions and the Heterogeneity of Variance**

We assumed that observed and reported data for COVID-19 for each country as well as for NPIs implemented by governments to prevent, control, and mitigate local outbreaks and virus circulation, cannot be defined as truly independent to one another. Actually, as COVID-19 is an infectious disease highly contagious, local and cross-border movement of people in neighbouring countries are likely to drive the emergence and the speed of outbreaks. Furthermore, as for the European Union, the timeliness and quality of implementation and adoption of NPIs (e.g. quarantine, contact tracing, school closure, work from home policies, and lock-downs) are shared and can be driven to outbreaks occurring in countries with geographical proximity. Therefore, we computed cluster-robust standard errors (using a clustered sandwich estimator) for the significant predictors of log-transformed COVID-19 deaths per million people as well as OLS standard errors in the final OLS regression model, to check for intragroup correlation between countries. The issue of heterogeneity of variance was explored comparing robust standard errors (using the Huber sandwich estimator) with the regular OLS ones. Results are reported only for the final list of COVID-19 deaths predictors, both adjusted and unadjusted by response measures (**Tables** **S5** and **S6**, respectively).

**Model Diagnostics**

The final OLS regression model adjusted for response measures was tested to check if all the linear regression assumptions were met. In particular, we tested for (i) linear association, (ii) multicollinearity, (iii) normal distribution of residuals, (iv) homoscedasticity. Moreover, we explored the presence of problematic influential residuals.

Linear association was tested in the univariate analysis as displayed in **Table 1** and in **Figures S2-S5**, on which the relationships between the log of COVID-19 deaths per million people and quantitative covariates included in the final model were graphed.

A variance inflation factor (VIF) above 5 was considered as the threshold for multicollinearity. In **Table S7**, we reported VIFs for the final model which shows no problematic multicollinearity.

Studentized (jackknifed) residuals were computed to check their normal distribution and homogeneity of variance (i.e., homoscedasticity). Both assumptions were tested graphically and using statistical methods. Firstly, residual-versus-fitted plot was graphed. The result showed homoscedasticity which was later confirmed using both Breusch-Pagan/Cook-Weisberg (p=0.110) test. Secondly, we tested the normal distribution of residuals. Quantiles of residuals against quantiles of normal distribution, standardized normal probability, and univariate Kernel density estimation plot for residuals plots were graphed. The results showed left-skewed distribution of residuals which was later confirmed using the Shapiro-Wilk test (p<0.001). Four countries were identified as probable mild outliers (Lawrence C. Hamilton test for detecting the number of mild and severe outliers depending on the inter-quartile range of the distribution of residuals [[www.stata.com/stb/stb3/sed4/iqr.hlp](http://www.stata.com/stb/stb3/sed4/iqr.hlp)]).

In **Figure S6**, studentized residuals are graphed against fitted values resulted from the final OLS model without removing outlier observations, as reported in **Table S6**.

**Identification and exclusion of influential outliers**

After computing studentized residuals, we used the Grubbs’ test to identify and remove outliers (p<0.050). The distribution of residuals and outlier observation were graphed using a boxplot for skewed or heavy-tailed distributions (**Figure S7**). Three countries were identified as possible outliers and, therefore, removed: Mongolia (r=-3.832), Papua New Guinea (r=-3.718), and Thailand (r=-3.451).

**Further adjustments**

Data on COVID-19 tests were very scarce and heterogeneously reported with some countries reporting “no. of individuals tested” and others providing information on the “no. of COVID-19 tests performed”. When incorporating this variable in either the linear OLS regression models or the robust regression models, the number of observations halved, from 136 observations to 57 countries included in the models.

Conversely, models encompassing the date of the 1^st^ COVID-19 case reported by each country were more conservative regarding the number of observations that dropped from 136 to 132. We have, therefore, added this variable in the analyses described in the next paragraph.

**Robust regression models and comparison with linear OLS regression models**

We developed a forest plot comparing the coefficients resulting from the linear OLS regression models and robust regression models with and without further adjusting for the log of the days between the detection and reporting of the 1^st^ COVID-19 case in each country and the censoring date (i.e., 31^st^ December 2021), without controlling for the geographical autocorrelation (**Figure S8**).

The models controlled for the date of detection of the 1^st^ COVID-19 case within countries, however, do not add additional information to the model presented in the main results as these already include the timeliness of the implementation of countries’ responses (i.e., stay-at-home requirements). Controlling for this variable would be reasonable if SARS-CoV-2 had a winter-seasonal pattern in the early phases of the pandemic, which is arguable having the majority of the world population susceptible.

**Cluster analysis results**

Three clusters were defined based on the BIC for models based on the final multivariable OLS model (**Table S3**), with VEI the most performing model (n=43; BIC=-1,832.2)

Clusters no. 1 (n=18) and 2 (n=9) encompass countries arbitrarily included in *the West* and cluster no. 3 (n=16) includes countries defined as *the East* (i.e., Cambodia, China, Indonesia, Japan, Laos, Malaysia, Mongolia, Myanmar, Papua New Guinea, Singapore, Thailand, Timor-Leste, and Viet Nam), except for Norway which is “mis-clustered” into *the East* and for Australia and New Zealand which are included in cluster no. 1 although arbitrarily classified as a country of *the East* as per the **Table S2**. Norway has most of the attributes of western countries, the only variable differing from the others classified in *the West* is the timeliness of the implementation of stay-at-home requirements. When excluding response covariates in the cluster analysis, Norway is classified among others western countries (results not displayed). Australia and New Zealand are countries with several attributes peculiar to western ones such as the prevalence of obesity in the country is 29% and 30.8%, respectively, vs. a median value of 6.1% for *the East* – when excluding Australia and New Zealand – and a higher state of democracy for Australia and New Zealand (democracy index of 9.1 and 9.3, respectively) compared to the rest of the region (democracy index of 6.4)

Noteworthy, this clustering differentiates countries from Western Europe (i.e., Austria, Belgium, Denmark, Estonia, Finland, France, Germany, Greece, Ireland, Italy, Netherland, Portugal, Spain, Sweden, and the United Kingdom) and North America (i.e., United States of America and Canada) from those of the Eastern Europe (i.e., Bulgaria, Croatia, Czech Republic, Hungary, Latvia, Lithuania, Polonia, Romania, Slovakia).

# **Supplemental Tables**

***Table S3* List of countries ≥ 1 million population**

|  | Cases per million | Deaths per million | Case-fatality ratio (%) |
| --- | --- | --- | --- |
| Afghanistan | 1323.612 | 56.283 | 4.25 |
| Albania | 20264.09 | 410.383 | 2.03 |
| Algeria | 2271.554 | 62.849 | 2.77 |
| Angola | 534.073 | 12.323 | 2.31 |
| Argentina | 35966.06 | 956.837 | 2.66 |
| Armenia | 53795.62 | 952.675 | 1.77 |
| Australia | 1114.711 | 35.647 | 3.20 |
| Austria | 40062.07 | 690.842 | 1.72 |
| Azerbaijan | 21569.8 | 260.475 | 1.21 |
| Bahrain | 54463.99 | 206.866 | 0.38 |
| Bangladesh | 3118.052 | 45.899 | 1.47 |
| Belarus | 20560.63 | 150.699 | 0.73 |
| Belgium | 55782.35 | 1684.957 | 3.02 |
| Benin | 268.164 | 3.629 | 1.35 |
| Bolivia (Plurinational State of) | 13717.43 | 785.143 | 5.72 |
| Bosnia and Herzegovina | 33828.48 | 1234.449 | 3.65 |
| Botswana | 6295.647 | 17.86 | 0.28 |
| Brazil | 36112.13 | 917.151 | 2.54 |
| Bulgaria | 29109.54 | 1090.316 | 3.75 |
| Burkina Faso | 320.859 | 4.066 | 1.27 |
| Burundi | 68.793 | 0.168 | 0.24 |
| Cambodia | 22.609 | 0 | 0 |
| Cameroon | 989.872 | 16.876 | 1.70 |
| Canada | 15484.25 | 417.623 | 2.67 |
| Central African Republic | 1027.586 | 13.044 | 1.27 |
| Chad | 128.639 | 6.331 | 4.92 |
| Chile | 31856.37 | 868.792 | 2.73 |
| China | 66.672 | 3.322 | 4.98 |
| Colombia | 32285.41 | 849.264 | 2.63 |
| Congo | 1287.945 | 19.572 | 1.52 |
| Costa Rica | 33238.56 | 428.926 | 1.29 |
| Croatia | 51357.67 | 954.871 | 1.86 |
| Cuba | 1047.356 | 12.89 | 1.23 |
| Czechia | 67108.24 | 1081.335 | 1.61 |
| Côte d'Ivoire | 852.596 | 5.194 | 0.61 |
| Democratic Republic of the Congo | 197.161 | 6.599 | 3.35 |
| Denmark | 28333.95 | 224.094 | 0.79 |
| Dominican Republic | 15743.59 | 222.531 | 1.41 |
| Ecuador | 12045.08 | 795.44 | 6.60 |
| Egypt | 1349.126 | 74.569 | 5.53 |
| El Salvador | 7085.812 | 204.588 | 2.89 |
| Equatorial Guinea | 3761.266 | 61.298 | 1.63 |
| Eritrea | 372.206 | 0.846 | 0.08 |
| Estonia | 21100.02 | 172.63 | 0.82 |
| eSwatini | 8066.101 | 176.699 | 2.19 |
| Ethiopia | 1080.899 | 16.727 | 1.55 |
| Finland | 6516.664 | 101.25 | 1.55 |
| France | 41022.25 | 992.118 | 2.42 |
| Gabon | 4300.166 | 28.755 | 0.67 |
| Gambia | 1571.174 | 51.31 | 3.27 |
| Georgia | 57009.28 | 627.949 | 1.10 |
| Germany | 21012.62 | 403.311 | 1.92 |
| Ghana | 1762.659 | 10.781 | 0.61 |
| Greece | 13321.43 | 464.163 | 3.48 |
| Guatemala | 7703.468 | 268.649 | 3.49 |
| Guinea | 1044.865 | 6.168 | 0.59 |
| Guinea-Bissau | 1245.936 | 22.866 | 1.84 |
| Haiti | 876.91 | 20.697 | 2.36 |
| Honduras | 12300.03 | 316.015 | 2.57 |
| Hungary | 33385.33 | 987.231 | 0 |
| India | 7439.595 | 107.781 | 2.96 |
| Indonesia | 2717.125 | 80.936 | 1.45 |
| Iran (Islamic Republic of) | 14586.25 | 657.472 | 2.98 |
| Iraq | 14799.95 | 318.553 | 4.51 |
|  | **Cases per million** | **Deaths per million** | **Case-fatality ratio (%)** |
| Ireland | 18587.04 | 453.036 | 2.44 |
| Israel | 48900.7 | 384.147 | 0.79 |
| Italy | 34851.18 | 1226.542 | 3.52 |
| Jamaica | 4331.747 | 101.987 | 2.35 |
| Japan | 1864.466 | 26.029 | 1.40 |
| Jordan | 28863.07 | 375.767 | 1.30 |
| Kazakhstan | 10715.19 | 147.044 | 1.37 |
| Kenya | 1793.857 | 31.057 | 1.73 |
| Kuwait | 35260.93 | 218.707 | 0.62 |
| Kyrgyzstan | 12420.54 | 207.689 | 1.67 |
| Laos | 5.635 | 0 | 0 |
| Latvia | 21685.91 | 336.655 | 1.55 |
| Lebanon | 26592.12 | 215.078 | 0.81 |
| Lesotho | 1444.275 | 23.807 | 1.65 |
| Liberia | 351.743 | 16.411 | 4.67 |
| Libya | 14593.63 | 215.098 | 1.47 |
| Lithuania | 52145.42 | 659.738 | 1.04 |
| Madagascar | 639.702 | 9.425 | 1.47 |
| Malawi | 344.12 | 9.88 | 2.87 |
| Malaysia | 3491.627 | 14.552 | 0.42 |
| Mali | 350.109 | 13.283 | 3.79 |
| Mauritania | 3089.258 | 74.629 | 2.42 |
| Mauritius | 414.384 | 7.863 | 1.90 |
| Mexico | 11060.76 | 975.757 | 8.82 |
| Mongolia | 372.145 | 0.305 | 0.08 |
| Morocco | 11898.85 | 200.16 | 1.68 |
| Mozambique | 596.44 | 5.311 | 0.89 |
| Myanmar | 2290.58 | 49.293 | 2.15 |
| Namibia | 9422.193 | 80.68 | 0.86 |
| Nepal | 8943.773 | 63.699 | 0.71 |
| Netherlands | 47177.59 | 672.605 | 1.43 |
| New Zealand | 448.34 | 5.184 | 1.16 |
| Nicaragua | 912.665 | 24.907 | 2.73 |
| Niger | 137.276 | 4.296 | 3.13 |
| Nigeria | 424.989 | 6.253 | 1.47 |
| North Macedonia | 39997.02 | 1201.413 | 3.00 |
| Norway | 9143.108 | 80.424 | 0.88 |
| Oman | 25235.27 | 293.54 | 1.16 |
| Pakistan | 2182.864 | 46.068 | 2.11 |
| Panama | 57196.59 | 932.147 | 1.63 |
| Papua New Guinea | 87.18 | 1.006 | 1.15 |
| Paraguay | 15132.36 | 317.139 | 2.10 |
| Peru | 30787.99 | 1142.793 | 3.71 |
| Philippines | 4326.148 | 84.358 | 1.95 |
| Poland | 34213.85 | 754.467 | 2.21 |
| Portugal | 40569.77 | 677.277 | 1.67 |
| Qatar | 49923.98 | 85.038 | 0.17 |
| Republic of Korea | 1204.798 | 17.886 | 1.48 |
| Republic of Moldova | 35899.68 | 739.967 | 2.06 |
| Romania | 32865.86 | 819.589 | 2.49 |
| Russian Federation | 21429.81 | 385.591 | 1.80 |
| Rwanda | 647.226 | 7.103 | 1.10 |
| Saudi Arabia | 10419.44 | 178.751 | 1.72 |
| Senegal | 1143.101 | 24.486 | 2.14 |
| Serbia | 49660.99 | 471.887 | 0.95 |
| Sierra Leone | 327.317 | 9.527 | 2.91 |
| Singapore | 10016.34 | 4.957 | 0.05 |
| Slovakia | 32885.48 | 391.601 | 1.19 |
| Slovenia | 58757.09 | 1297.301 | 2.21 |
| Somalia | 296.604 | 8.18 | 2.76 |
| South Africa | 17824.72 | 480.014 | 2.69 |
| South Sudan | 317.857 | 5.628 | 1.77 |
| Spain | 41242.09 | 1087.311 | 2.64 |
| Sri Lanka | 2022.066 | 9.527 | 0.47 |
| State of Palestine | 27052.1 | 274.434 | 1.01 |
| Sudan | 581.538 | 33.478 | 6.30 |
| Sudan | 581.538 | 33.478 | 6.30 |
| Sweden | 43307.98 | 864.122 | 2.00 |
| Switzerland | 52260.65 | 883.343 | 1.69 |
| Syrian Arab Republic | 653.347 | 40.627 | 6.22 |
|  | **Cases per million** | **Deaths per million** | **Case-fatality ratio (%)** |
| Taiwan | 33.548 | 0.294 | 0.88 |
| Tajikistan | 1394.055 | 9.436 | 0.68 |
| Thailand | 102.622 | 0.903 | 0.88 |
| Timor-Leste | 33.373 | 0 | 0 |
| Togo | 438.835 | 8.214 | 1.87 |
| Trinidad and Tobago | 5109 | 90.747 | 1.78 |
| Tunisia | 11772.95 | 395.647 | 3.36 |
| Turkey | 26187.77 | 247.584 | 0.95 |
| Uganda | 769.9 | 5.487 | 0.71 |
| Ukraine | 24854.87 | 440.872 | 1.77 |
| United Arab Emirates | 21012.5 | 67.641 | 0.32 |
| United Kingdom | 36770.98 | 1084.495 | 2.95 |
| United Republic of Tanzania | 8.521 | 0.352 | 4.13 |
| United States of America | 60609.49 | 1065.638 | 1.73 |
| Uruguay | 5503.887 | 52.105 | 0.95 |
| Uzbekistan | 2302.415 | 18.345 | 0.80 |
| Venezuela (Bolivarian Republic of) | 3993.467 | 36.151 | 0.91 |
| Viet Nam | 15.051 | 0.36 | 2.39 |
| Yemen | 70.375 | 20.452 | 29.06 |
| Zambia | 1127.342 | 21.105 | 1.87 |
| Zimbabwe | 932.993 | 24.423 | 2.62 |

***Table S4* multiple linear regression explorative sub-models based on significant variables from domain-specific stepwise backward selection.**

|  | **Sub-model Domain no. 1** |  | **Sub-model Domain no. 2** |  | **Sub-model Domain no. 3** |  |
| --- | --- | --- | --- | --- | --- | --- |
|  | *b* (95% CI) |  | *b* (95% CI) |  | *b* (95% CI) |  |
| Constant | 1.866 (1.758 to 1.973) | **^*^** | 1.989 (1.852 to 2.124) | **^*^** | 1.872 (1.753 to 1.992) | **^*^** |
| Population density (km^-3^) | 0.120 (-0.258 to 0.018) | **^*^** |  |  |  |  |
| Median age (years) | 0.366 (0.239 to 0.492) | **^*^** |  |  |  |  |
| Obesity prevalence (%) | 0.397 (0.269 to 0.524) | **^*^** |  |  |  |  |
| Medical doctors’ density (per 10,000 population) |  |  | 0.336 (0.189 to 0.482) | **^*^** |  |  |
| GHS index (%) |  |  | 0.357 (0.185 to 0.528) | **^*^** |  |  |
| Previous SARS cases (‘Yes’) |  |  | -0.618 (-0.997 to -0.239) | **^*^** |  |  |
| EIU democracy index (0-10) |  |  |  |  | 0.242 (0.063 to 0.420) | ^*^ |
| UHC service coverage (%) |  |  |  |  | 0.517 (0.355 to 0.679) | ^*^ |
| Current health expenditure (% GDP) |  |  |  |  | 0.115 (-0.024 to 0.255) |  |
| Political Stability and Absence of Violence (%) |  |  |  |  | -0.264 (-0.445 to  -0.084) | **^*^** |
| **Model statistics** |  |  |  |  |  |  |
| Observations | 145 |  | 148 |  | 145 |  |
| R^2^ | 0.512 |  | 0.370 |  | 0.398 |  |
| Adjusted R^2^ | 0.501 |  | 0.357 |  | 0.380 |  |
| Residual SE | 0.65 |  | 0.74 |  | 0.73 |  |
| F statistic (df) | 49.20 (3, 141) |  | 28.17 (3, 144) |  | 23.09 (4, 140) |  |
| p-value | p<0.001 |  | p<0.001 |  | p<0.001 |  |
| AIC | 291.44 |  | 333.09 |  | 323.51 |  |
| Sample-size adj. BIC | 303.35 |  | 345.08 |  | 338.40 |  |
|  |  |  |  |  |  |  |
| ^*^ Significant p-values (α ≤ 0.100) for b coefficients of the specific multivariate OLS regression sub-models against COVID-19 deaths per million (logged). ^†^ Log-transformed to normalize the distribution frequency.  Abbreviations: EIU=Economist intelligence unit; GDP=gross domestic product; GHS=Global Health Security; SARS=severe acute respiratory syndrome; UHC=universal health coverage. | | | | | | |

***Table S5* multiple linear regression final models without removing possible outliers nor adjusting for response measures.**

|  | **Final model** | | **Correction for spatial autocorrelation** | **Robust variance estimates model** |
| --- | --- | --- | --- | --- |
|  | *b* (95% CI)  p-value | | *b* (95% CI)  p-value | *b* (95% CI)  p-value |
| Constant | 1.951 (1.841 to 2.062) p<0.001 | | 1.951 (1.842 to 2.060) p<0.001 | 1.951 (1.854 to 2.049)  p<0.001 |
| Median age (years) | 0.430 (0.272 to 0.588) p<0.001 | | 0.430 (0.203 to 0.658) p=0.001 | 0.430 (0.247 to 0.613) p<0.001 |
| Obesity prevalence (%) | 0.399 (0.282 to 0.515) p<0.001 | | 0.399 (0.247 to 0.548) p<0.001 | 0.399 (0.280 to 0.517) p<0.001 |
| Previous cases of SARS (‘Yes’) | -0.438 (-0.723 to -0.152) p=0.003 | | -0.438 (-0.920 to 0.045) p=0.072 | -0.438 (-0.796 to -0.079) p=0.017 |
| EIU democracy index (0-10) | 0.290 (0.144 to 0.436) p<0.001 | | 0.290 (0.036 to 0.544) p=0.028 | 0.290 (0.144 to 0.435) p=0.001 |
| Political Stability and Absence of Violence (%) | -0.293 (-0.447 to -0.138) p<0.001 | | -0.293 (-0.513 to -0.072) p=0.013 | -0.293 (-0.468 to -0.117) p<0.001 |
| **Model statistics** |  | |  |  |
| Observations | 146 | 146 | | 146 |
| R^2^ | 0.583 | 0.583 | | 0.583 |
| Adjusted R^2^ | 0.568 | 0.568 | | - |
| Residual SE | 0.61 | 0.61 | | 0.61 |
| F statistic (df) | 39.16 (5, 140) | | 76.47 (5, 14) | 49.16 (5, 140) |
| p-value | p<0.001 | p<0.001 | | p<0.001 |
| AIC | 273.31 | 273.31 | | 273.31 |
| Sample-size adj. BIC | 291.22 | 291.22 | | 291.22 |
|  |  | |  |  |
| Regions are controlled as sampling clusters and the clustering effect was accounted for using cluster-robust standard errors (the clustered sandwich estimator).  Abbreviations: EIU=Economist intelligence unit; SARS=Severe acute respiratory syndrome. | | | | |

***Table S6* multiple linear regression final models without removing possible outliers, adjusted for response measures.**

|  | **Final model** | | **Correction for spatial autocorrelation** | **Robust variance estimates model** |
| --- | --- | --- | --- | --- |
|  | *b* (95% CI)  p-value | | *b* (95% CI)  p-value | *b* (95% CI)  p-value |
| Constant | 1.944 (1.837 to 2.051)  p<0.001 | | 1.944 (1.852 to 2.037)  p<0.001 | 1.944 (1.853 to 2.035)  p<0.001 |
| Median age (years) | 0.399 (0.249 to 0.549)  p<0.001 | | 0.399 (0.163 to 0.635)  p=0.003 | 0.399 (0.215 to 0.582)  p<0.001 |
| Obesity prevalence (%) | 0.343 (0.230 to 0.456)  p<0.001 | | 0.343 (0.189 to 0.497)  p<0.001 | 0.343 (0.226 to 0.460)  p<0.001 |
| Previous cases of SARS (‘Yes’) | -0.478 (-0.754 to -0.202)  p<0.001 | | -0.478 (-0.958 to 0.002)  p=0.051 | -0.478 (-0.835 to -0.121)  p=0.009 |
| EIU democracy index (0-10) | 0.324 (0.183 to 0.465)  p<0.001 | | 0.324 (0.107 to 0.540)  p=0.006 | 0.324 (0.176 to 0.472)  p<0.001 |
| Political Stability and Absence of Violence (%) | -0.255 (-0.405 to -0.106)  p<0.001 | | -0.255 (-0.431 to -0.080)  p=0.007 | -0.255 (-0.432 to -0.079)  p=0.005 |
| Average stringency index (%) | 0.189 (0.064 to 0.315)  p=0.003 | | 0.189 (0.035 to 0.344)  p=0.020 | 0.189 (0.051 to 0.328)  p=0.008 |
| Timeliness of stay-at-home requirements (days) | -0.054 (-0.164 to 0.055)  p=0.328 | | -0.054 (-0.181 to 0.072)  p=0.371 | -0.054 (-0.150 to 0.041)  p=0.261 |
| **Model statistics** |  | |  |  |
| Observations | 136 | 136 | | 136 |
| R^2^ | 0.617 | 0.617 | | 0.617 |
| Adjusted R^2^ | 0.596 | 0.596 | | - |
| Residual SE | 0.559 | 0.559 | | 0.559 |
| F statistic (df) | 29.50 (7, 128) | | 92.23 (7, 14) | 45.16 (7, 128) |
| p-value | p<0.001 | p<0.001 | | p<0.001 |
| AIC | 235.32 | 235.32 | | 235.32 |
| Sample-size adj. BIC | 258.62 | 258.62 | | 258.62 |
|  |  | |  |  |
| Regions are controlled as sampling clusters and the clustering effect was accounted for using cluster-robust standard errors (the clustered sandwich estimator).  Abbreviations: EIU=Economist intelligence unit; SARS=Severe acute respiratory syndrome. | | | | |

# **Supplemental Figures**

| 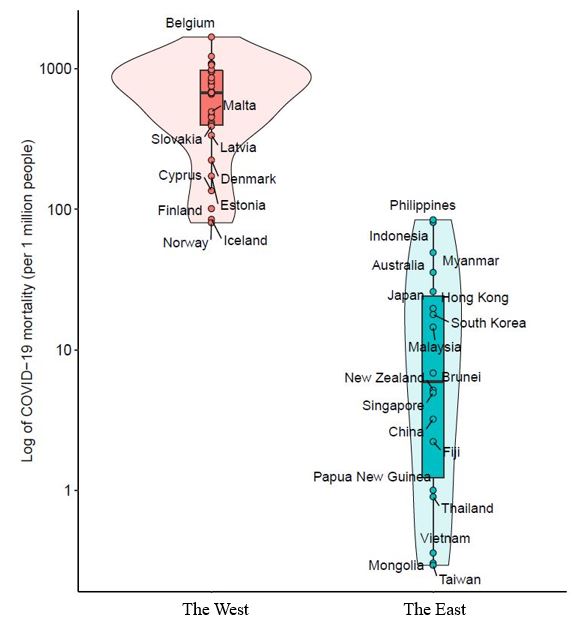 |
| --- |
| ***Figure S1* The different COVID-19 mortality (per 1 million population) in the West and in the East, during the year 2020.** |

| 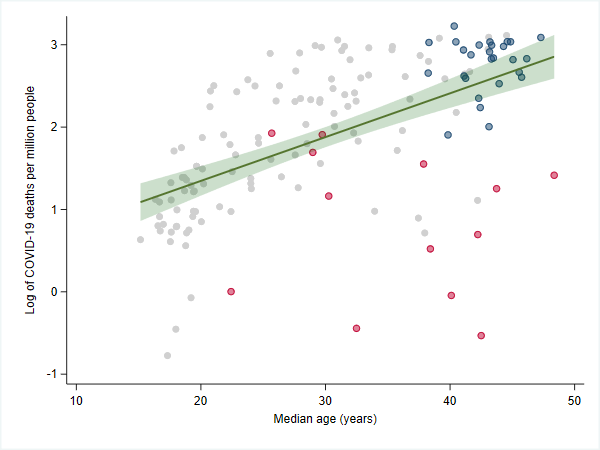 | N = 151  Adj. R^2^ = 0.305  r = 0.556  β = 0.506 (95%CI: 0.384 to 0.629)  p-value < 0.001 |
| --- | --- |
|  | 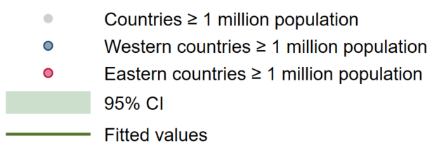 |
| ***Figure S2* Association between log of COVID-19 deaths per million people and countries’ population median age (years)** | |

| 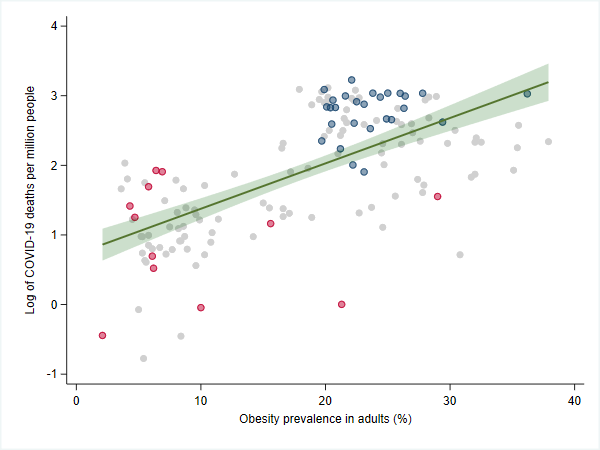 | N = 146  Adj. R^2^ = 0.424  r = 0.655  β = 0.596 (95%CI: 0.483 to 0.710)  p-value < 0.001 |
| --- | --- |
|  | 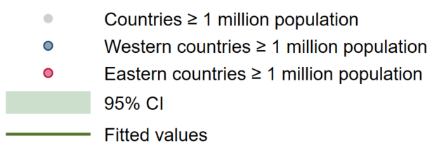 |
| ***Figure S3* Association between the log of COVID-19 deaths per million and obesity prevalence in adults (%)** | |

| 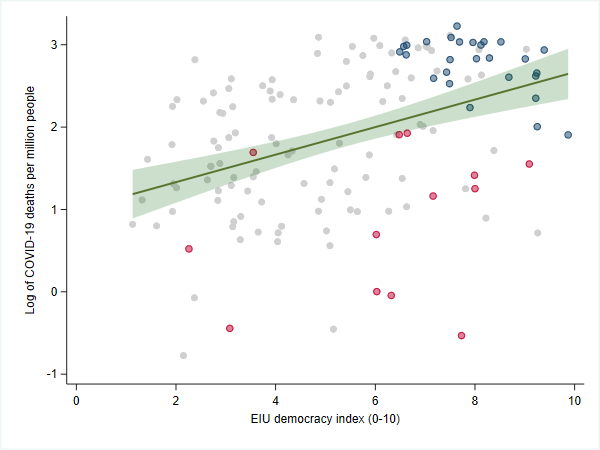 | N = 149  Adj. R^2^ = 0.159  r = 0.405  β = 0.370 (95%CI: 0.234 to 0.506)  p-value = 0.081 |
| --- | --- |
|  | 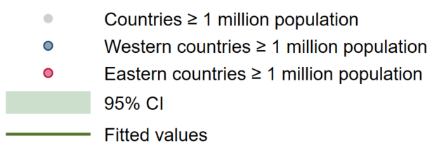 |
| ***Figure S4* Association between the log of COVID-19 deaths per million and EIU democracy index (0-10)** | |

| 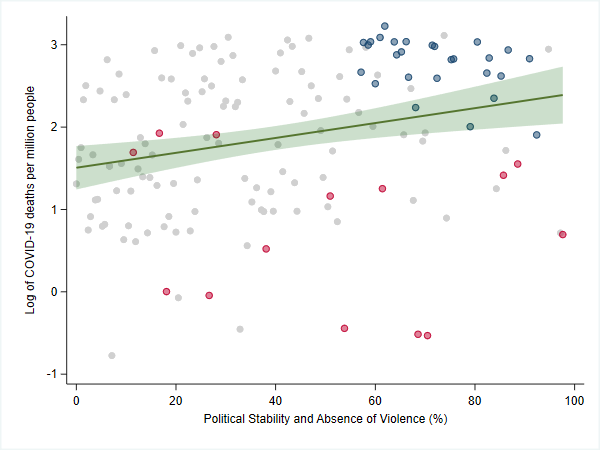 | N = 152  Adj. R^2^ = 0.066  r = 0.258  β = 0.238 (95%CI: 0.094 to 0.382)  p-value = 0.001 |
| --- | --- |
|  | 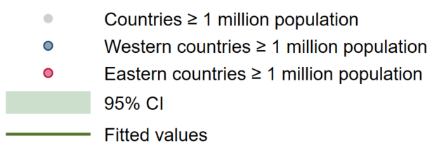 |
| ***Figure S5* Association between the log of COVID-19 deaths per million and Political Stability and Absence of Violence (%)** | |

# **Post-estimation diagnostics and robustness checks**

***Table S7* Variance inflation factors for independent variables used in the final model adjusted for response measures**

|  | **VIF** | **1/VIF** |
| --- | --- | --- |
| Median age (years) | 2.50 | 0.40 |
| Obesity prevalence (%) | 2.43 | 0.41 |
| Previous SARS cases (‘Yes’) | 2.17 | 0.46 |
| EIU democracy index (0-10) | 1.39 | 0.72 |
| Political Stability and Absence of Violence (%) | 1.31 | 0.76 |
| Avg stringency index (%) | 1.22 | 0.82 |
| Timeliness stay-at-home requirements (days) | 1.15 | 0.87 |
| **Mean VIF** | **1.74** |  |

| 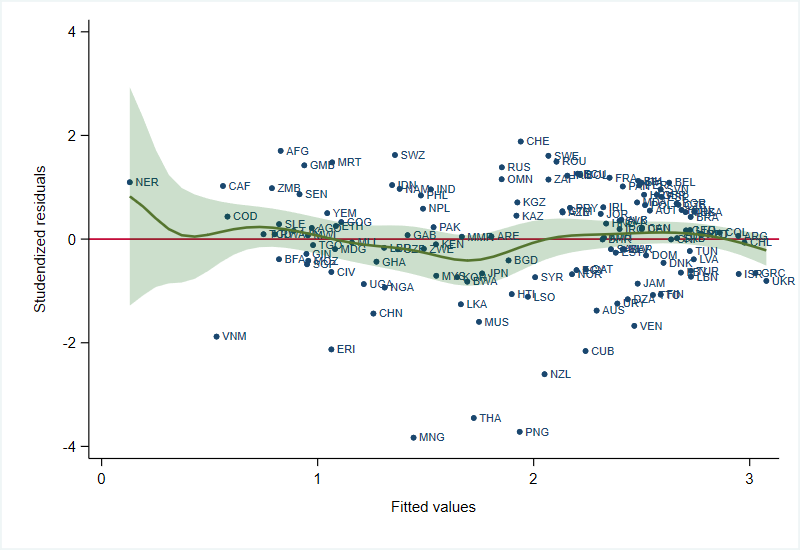 |
| --- |
| ***Figure S6* Relationship between predicted values (adjusted for response measures) and model residuals for COVID-19 deaths per million people**  Outliers are not excluded from this graph. |

| 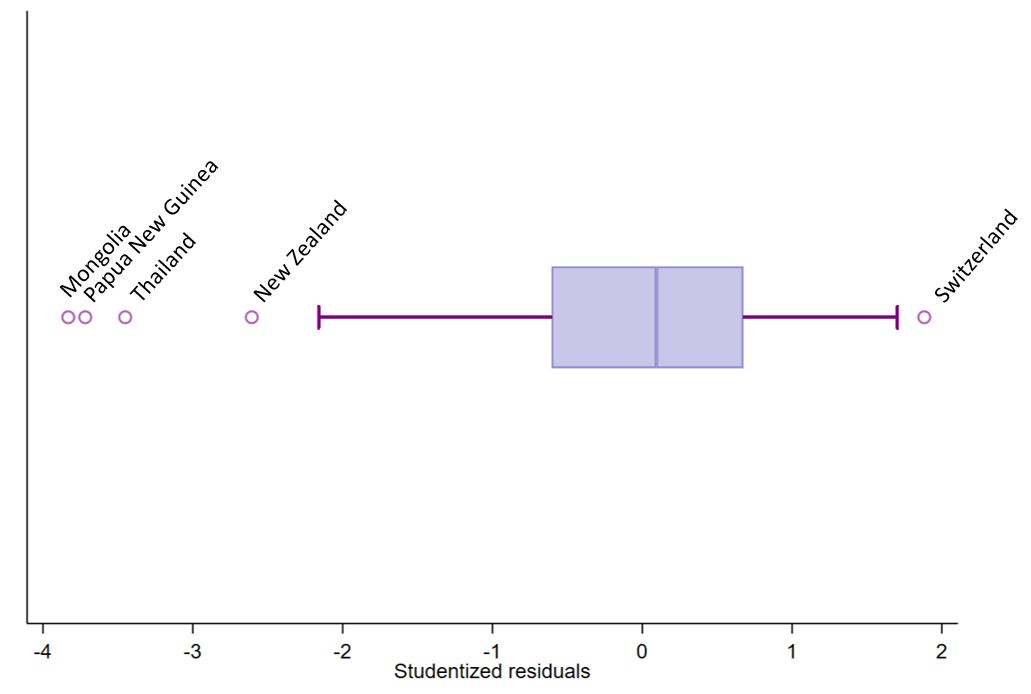 |
| --- |
| ***Figure S7* Boxplot for skewed or heavy-tailed distributions**  Labelled observations are those considered (mild) outliers based on their studentized residuals. |

| 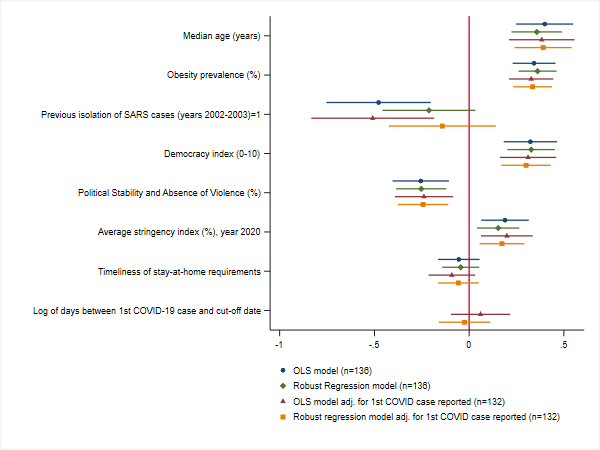 |
| --- |
| ***Figure S8* Forest plot comparing linear OLS regression models and robust regression models, with and without the period of reporting of the 1^st^ COVID-19 case.** |

| **(a)** | **(b)** |
| --- | --- |
| **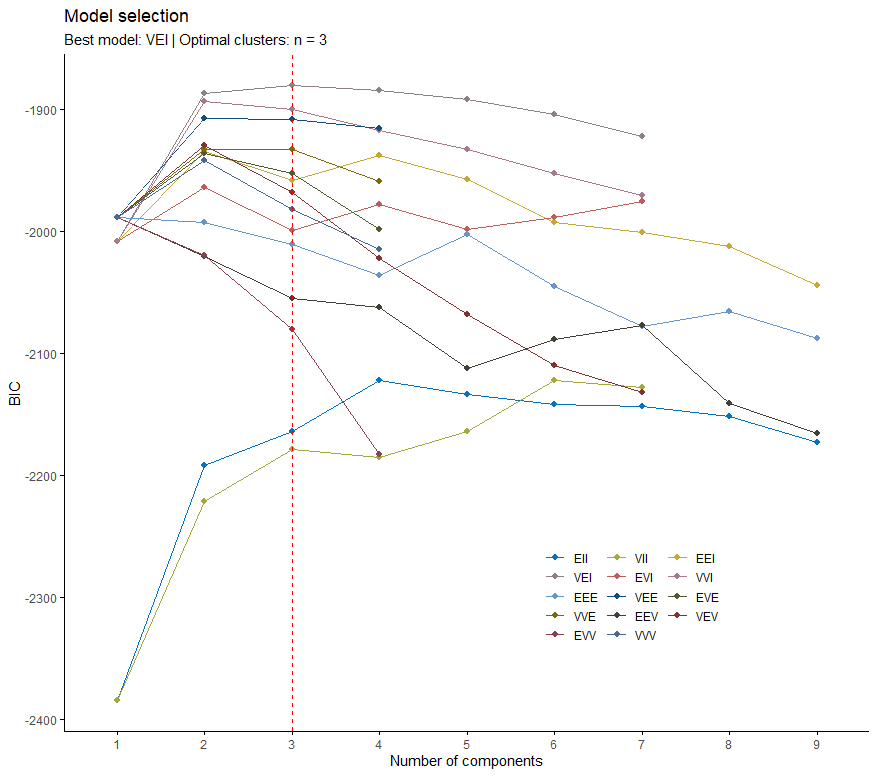** | **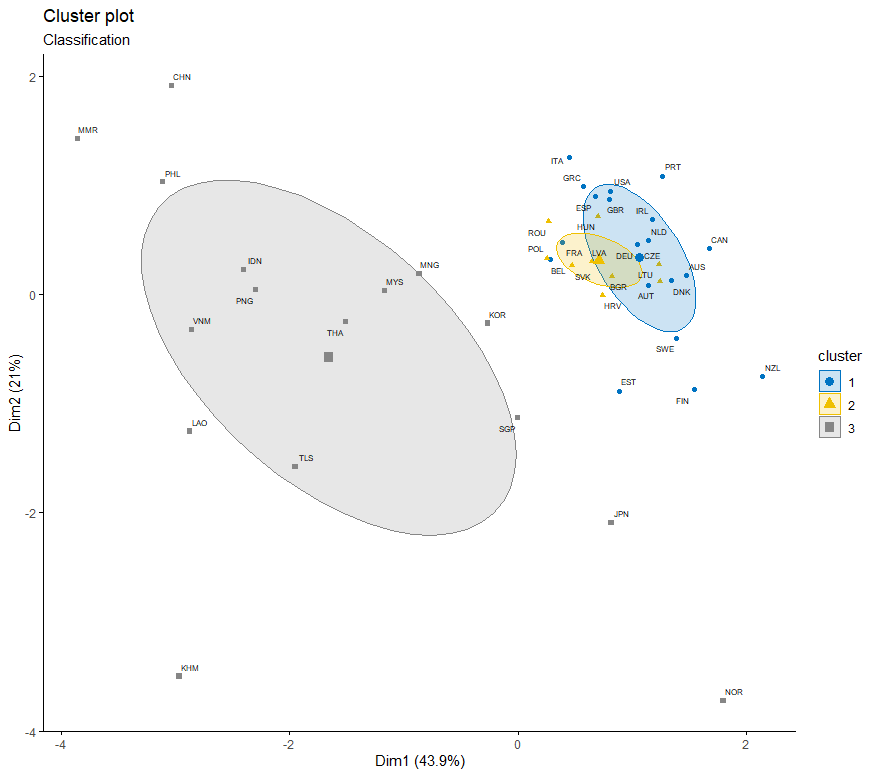** |
| ***Figure S9* Model-based clustering and classification estimation.**  **(a)** BIC values are computed for each model depending on the number of components (i.e., clusters) based on the final multivariable OLS model’s covariates (**Table 2**) by displaying the BIC traces for all the models considered ; **(b)** Cluster plot display the classification of the countries reported in **Table S2** in two main clusters. Most countries in clusters no. 1 (blue) and no. 2 (yellow) are those arbitrarily classified as included in *the West*, except for AUS (i.e., Australia) and NZL (i.e., New Zealand); whereas, countries in cluster no. 3 (grey) are those from *the East*, a part from NOR (i.e., Norway).  Analysis and plotting using *mclust* and *factorextra* packages in R (version 4.0.3). | |
